# Medical History from the Earliest Times

**Published:** 1892-06-11

**Authors:** E. T. Withington


### Medical History from the Earliest Times.

By E. T. Withington, M.A., M.B. Oxon.

### XII.—THE SUCCESSORS OF HIPPOCRATES.—THE DOGMATIC SCHOOL.

Lord Bacon's remark on the science of the ancients that "she was old enough to talk, but not old enough to bear children," may in some sense be applied to Greek medicine. Not that the science of Hippocrates and Herophilus, of Heraclides and Soranus, was sterile, far from it; some of her sons might have justified the boasts of the Mother of the Gracchi; but she certainly did an immense amount of talking. The ancient Greek loved talking; his mind was more philosophical than scientific, and he preferred to speculate on things in general rather than to investigate particular facts. His failure in medicine, so far as we dare call it a failure, was thus due to causes the very opposite to those which produced the downfall of the art in ancient Egypt. A Hippocratic writer had said, "The physician who is also a philosopher is godlike." This became the motto of the Dogmatic school, was made the excuse for an immense amount of useless speculation, and was finally taken as the text of a special treatise by Galen himself. But there were two more direct sources for those floods of theory which spoilt the fair promise of the Hippocratic harvest—the Court physicians and the philosophy of Plato.

The supposed founders of dogmatism, the sons of Hippocrates, Thessalus and Draco, seem to have been both employed at the Macedonian Court, and the interest taken in medicine by the successors of Alexander is notorious. We need only mention Attalus of Pergamus, who planted the first poison garden, and invented a lead plaster; Nicomedes, of Bithynia; Mithridates, of Pontus, the most famous of toxicologists; and, above all, the Greek Kings of Egypt. All these monarchs seem to have delighted in medical discussions, and doubtless those physicians who could bring forward the most numerous and plausible theories would be most likely to obtain their favour.

A still more important influence was that of Plato, who, though doubtless the noblest of philosophers, has hardly deserved well of the profession of medicine. A Court physician, whose livelihood depended on his being always able to render a reason to an inquisitive but royal amateur, may be pardoned if he sometimes went to his imagination for his facts; but when a philosopher, whose only object is the discovery of truth, and whose great Master was never weary of asserting his own ignorance, spins a huge cobweb of absurdities out of his inner consciousness, and imposes it upon mankind as the reality of nature he is less excusable. Plato had a low opinion of physicians, and declared that if they could not cure their patients quickly they were worse than useless, for they only prolonged lives worthless to the State. By his philosophy he added injury to the insult, and we shall find the Platonic physiology, sometimes in its own shape, sometimes in the yet more ghostly form of neoplatonism, turning up again and again as the evil genius of the healing art. Few now readthe "Timæus, but," next to the Homeric poems, it was probably the most popular of Greek writings; it had countless commentators; Cicero himself translated it into Latin, and together with the Hippocratic treatise, "On the Nature of Man," it seems to have formed the physiological textbook of the dogmatic physicians. Nor is it without value, even from a medical standpoint; Plato was acquainted with the writings of Hippocrates, and recognises the healing power of nature, declaring that "every form of disease is in a manner akin to the living being, whose complex frame has an appointed time of life," and that, in treatment, regimen is always to be preferred to drugs; but we need not attempt an abstract of the work, for it can be read by all in the English of Professor Jowett. The treatise "On the Nature of Man" is ascribed by Aristotle to Polybus, son-in-law of Hippocrates, and contains the humoral pathology in its most typical form, but an account of these doctrines will be best given when we come to speak of Galen, by whom the dogmatic theories were pruned into a definite system. Meanwhile, we may take the following description of medical dogmatism from the writings of our own Cullen: "Every wise physician is a dogmatist, but a dogmatic physician is one of the most absurd animals that lives. We say he is a dogmatist in physic who employs his reason, and from some acquaintance with the nature of the human body thinks he can throw some light upon diseases, and ascertain the proper methods of cure. On the other hand, I call him a dogmatical physician who is very ready to assume opinions and to be prejudiced in favour of them, and to retain and assert very tenaciously and with too much confidence the opinions and prejudices which he has already taken up in common life as in the study of the sciences."

Galen gives as the leaders of the Dogmatic, or as he prefers to call it, the Rational school, Hippocrates, Diocles of Carystus, Praxagoras of Cos, and the great Alexandrine anatomists. Diocles was classed in antiquity second both in age and rank to Hippocrates, but, unfortunately, we know little about him. He saw the danger of too much theory, and warned his colleagues against trying to explain everything; he devoted much time to anatomy, probably that of animals only, and carefully investigated the development of the embryo. Three inventions long survived under his name: a bandage for the head, a surgical instrument which we shall notice later on, and a remedy for toothache containing opium, galbanum, and pepper. He further distinguished pleurisy from pneumonia, and declared that fever was not a disease, but a symptom.

Praxagoras of Cos, the tutor of Herophilus, was a true dogmatist, and maintained the existence of no less than eleven different humours. He is also said to have first distinguished the arteries from the veins, and to have asserted that they contain air only, though both the discovery and the theory have been claimed to be of older date, and, as we have seen, were not unknown to the Egyptians. Of special interest is his treatment of intestinal obstruction, for which Diocles had proposed lead pills, apparently on the same principle that has caused metallic mercury to be given in recent times. Caelius Aurelianus tells us that after the failure of purgatives, enemata, emetics, and rectal injections of air, Praxagoras recommended massage of the abdomen, and finally laparotomy, "*dividendum ventrem*," removal of the obstruction and suture of the intestine, "*atque detracto stercore consuendum dicit* (*intestinum*)." It is, however, doubtful whether he actually performed the operation, or only recommended it as a counsel of perfection. Caelius further relates that Praxagoras had a slave who eat six pounds of bread daily without satisfying his appetite, which is probably the earliest recorded instance of bulimia, or abnormal hunger.

With all their faults, the Dogmatists were upon the right road; they saw that a science of medicine must be based upon physiology, and their error, which was almost unavoidable, was the attempt to erect a complete edifice before there were materials suitable or sufficient for the foundation. The theory of disease, or perverted vital action, forms the most difficult division of the most complex of the physical sciences, yet circumstances demanded that it should be attacked first, and the marvel is, not that the old physicians failed, but that they came so near to the truth. If the Dogmatists missed their mark, they at least aimed high, and it is they, and not the Empirics, the Methodics, or even the Eclectics, who are the truest sons of Hippocrates, the most legitimate fathers of modern medicine.

